# Chinese comprehenders’ interpretation of underinformativeness in L1 and L2 accented speech narratives

**DOI:** 10.3389/fpsyg.2023.1040162

**Published:** 2023-01-23

**Authors:** Yanrui Li, Shuo Feng

**Affiliations:** Institute of Linguistics and Applied Linguistics, Peking University, Beijing, China

**Keywords:** scalar implicature, ad hoc implicature, underinformative, accent, L2 speech narratives

## Abstract

Second language (L2) speakers with foreign accents are well-known to face disadvantages in terms of language processing; however, recent research has demonstrated possible social benefits for foreign-accented L2 speakers. While previous research has focused on the ways in which first language (L1) speakers of English comprehend L2 speech, the present article contributes to this line of research by exploring the ways in which comprehenders from a different culture and linguistic background perceive L2 speech narratives. This study investigates this issue by exploring how comprehenders with Mandarin Chinese as the first language interpret underinformative utterances containing scalar and ad hoc implicature in L1, accent-free L2, and foreign-accented L2 speech narratives. The sentence judgment task with a guise design used written sentences rather than oral utterances as stimuli in order to isolate the role of intelligibility factors. The results indicate that foreign accent confers social benefits on L2 speakers in that their omission of information in communication is tolerated and they are viewed as more likely to possess positive attributes. More importantly, we find that the bilingual characteristics of Chinese participants, as well as the different linguistic complexity of deriving scalar and ad hoc implicature, affect Chinese participants’ explanations of underinformative sentences of L2 speakers. This study contributes to our understanding of L2 language processing.

## 1. Introduction

Given the increasing number of bilinguals worldwide (Grosjean, 2010), a considerable number of people routinely communicate in a language that is not their native language, especially those who work or study overseas. Empirical studies have found learning difficulties, especially with aspect to the acquisition of second language (L2) phonology (Scovel, 1969; Flege et al., 1995; MacKay et al., 2001). It is unsurprising that many L2 speakers speak a second language with a foreign accent. The impact of foreign accent on communication and its broader social implications have been the focus of many studies dating back several decades. Specifically, foreign-accented utterances are found to be more difficult to understand and differences arise between comprehenders’ interpretation on first language (L1) speech and L2 foreign-accented speech (Davis et al., 2005; Lev-Ari and Keysar, 2012; Lev-Ari, 2015; Grey and van Hell, 2017). A group of researchers interpret these findings based on the “fluency-intelligibility” account which holds that a foreign accent requires additional processing efforts since it deviates from stored phonological representations in one’s native language norms and the linguistic disfluency such as distorted phonemes or prosody makes it costly to comprehend foreign-accented speech (e.g., Floccia et al., 2006; Lev-Ari and Keysar, 2010; Boduch-Grabka and Lev-Ari, 2021). An alternative explanation is the “expectation-based” account. Comprehenders have different expectations about L2 speakers such as their syntactic and semantic competence as compared to L1 speakers (e.g., Hanulíková et al., 2012). Cognitive scientists have noticed that expectation violation (as compared to the norm-consistent expectations of L1 speech) distract and redirect individuals’ attentional resources (Bartholow et al., 2001). As shown by recent research, expectations regarding speakers’ identity are drawn rapidly during sentence comprehension and influence the interpretation (Foucart et al., 2019).

To date, researchers interpret processing difference between L1 and L2 foreign-accented speech from either the “fluency-intelligibility” account or the “expectation-based” account but have not directly manipulated the intelligibility factor or the expectation factor. Using written stimuli in a reading task to examine how accented speech affects interpretation would be one possible way to disentangle the effects of intelligibility from effects of expectations since there is no processing cost from physical accent. In the presence of spoken language, listeners attend to and respond to both linguistic and nonlinguistic properties of speech. Listeners are not only sensitive to pitch, stress and other phonological cues, but also speaker-specific properties such as identity. Reading is a different task since individuals access phonological representations that are not physically available. However, some research has suggested that reading involves accessing both phonological representations and auditory word forms (Van Orden et al., 1990; Bentin and Ibrahim, 1996; Peng et al., 2004). Research investigating auditory imagery reveals that imagery for auditory events is similar to auditory perceptual experience. Auditory imagery is the subjective experience of hearing in the absence of auditory stimulation. For instance, research of auditory imagery for music showed that music imagery preserves detailed perceptual attributes of music such as pitch and timbre (Hubbard and Stoeckig, 1988; Crowder, 1989). Research using brain imaging technology reported that temporal lobe structures are activated in both auditory imagery events and actual auditory perception (Yoo et al., 2001; Halpern et al., 2004; Kraemer et al., 2005). More recent research found that when reading silently, phonological representations may include at least some perceptual aspects of actual speech (Abramson and Goldinger, 1997; Ashby and Clifton, 2005; Ashby, 2006). With explicit instructions, individuals with visual word forms can even form speaker-specific auditory images that are sustained in memory (Geiselman and Bellezza, 1977; Johnson et al., 1988).^1^

As such, the abovementioned findings allow reading to be a possible vehicle for examining the nature of how accented speech narratives affect interpretation without physical accent to process. This is important to note in that non-native accented speech is processed differently to the extent that a foreign accent taxes intelligibility and brings about additional processing load (Davis et al., 2005; Floccia et al., 2006). Comprehenders also have different expectations about non-native speech and they rely more on top-down extra-linguistic information (Niedzielski, 1999; Lev-Ari, 2015). Using written stimuli enabled us to manipulate the speaker identity (accented vs. non-accented) and made sure other properties of the linguistic stimuli (e.g., intelligibility) remained constant at the same time. By disentangling the effects of intelligibility from effects of expectations about non-native speech, we are able to focus on discussing the effects of expectations about speakers’ identity in comprehension. Therefore, any asymmetrical behavior in how sentences are interpreted across speakers’ conditions can be unambiguously attributed to different expectations about speaker identity. Using written stimuli, Fairchild and Papafragou (2018) and Fairchild et al. (2020) have suggested that even though there was no physical accent to process, non-native accent had a strong effect on social-pragmatic inferences and participants showed selective lenience toward non-native speakers. While the present study does not aim to examine how reading and hearing are similar or different, the results from Fairchild and colleagues and from the current study might be suggestive of strong lenience and robust social benefit on nonnative speakers in an auditory imaginary event. That is, even without physical accent to process, participants still access the abstract and perceptual nature of written stimuli by nonnative speakers during reading.

The current study aims to investigate interpretation of pragmatic inferences which offers a valuable opportunity to explore how speakers’ identity affects listeners’ interpretation. It is because pragmatic inferences go beyond the semantic meaning or grammatical structure of a sentence and include contextual enrichment that comprehenders derive what the speakers attempt to convey. Specifically, pragmatic inferences of (under) informative utterances are driven by expectations about how rational communication works in accordance with Cooperative Principle (Grice, 1975). Underinformative utterances violating the co-operative assumption and the Quantity Maxim (be informative) have been taken as speaker’s failure to be informative. The central role of speaker in deriving inference is also highlighted by the Rational Speech Act (RSA) framework (Frank and Goodman, 2012; Goodman and Frank, 2016) which is a Bayesian probabilistic account of modeling pragmatic inferences. Thus, the presence of speakers’ accent in deriving pragmatic inferences not only represents linguistic stimuli but also functions as the driving force for inferences (reasoning speakers’ failure to be informative) about the identity of the speaker. Furthermore, another important perspective in such communication is how comprehenders understand and perceive speakers who produce underinformative sentences. Research on person perception has shown that people evaluate others based on a variety of factors, such as the way they speak, facial expressions, in/out-group relation etc. What’s important to the current study is how people use variations in accent to make inferences about speakers and attribute judgments to the speakers. In constructing perception of others, warmth and competence are argued to be the two universal dimensions of human social cognition (Fiske et al., 2007). Empirical evidence shows that people often ascribe negative judgments to L2 speakers by judging them to be less honest and friendly or less intelligent and competent (Lambert et al., 1960; Bourdieu and Thompson, 1992; Gluszek and Dovidio, 2010; Lev-Ari and Keysar, 2010; Huang et al., 2013). This study contributes to research on person perception by investigating how Chinese comprehenders perceive L2 speakers with different degree of foreign accent based on pragmatic inferences, hoping to better understand the impact of foreign accent on communication and its broader social implications.

Additionally, previous studies (Gibson et al., 2017;Fairchild and Papafragou, 2018; Fairchild et al., 2020) have not addressed the question of whether the observed social benefits for L2 speakers are confined to a particular first language and cultural background. In these studies, L1 speech was produced by speakers whose L2 was English and was judged by L1 English speakers. As Fairchild et al. (2020, p. 8), noted, “it is important to consider whether different types of accents are equally likely to induce changes in pragmatic processing and how the listeners’ specific language background might affect the results.” Lastly, previous studies pertaining to L1 speaker’s comprehension of underinformativeness (Fairchild and Papafragou, 2018; Fairchild et al., 2020) have explored only one type of implicature in isolation, whether scalar implicature or ad hoc implicature. Theoretically speaking, although both types of implicature are closely related to the Quantity Maxim (Grice, 1989), their pragmatic inferences derive in a distinctively different manner: alternatives in scalar implicature are linguistically predetermined on a lexicalized scale, whereas alternatives in ad hoc implicature are determined by a particular situation in a specific context. Empirical data have found that differences in linguistic difficulty between scalar and ad hoc implicature affected interpretation (Papafragou and Musolino, 2003; Barner et al., 2011; Horowitz et al., 2017; Foppolo et al., 2021). Therefore, considering differences between these two types of implicatures in terms of linguistic complexity, they should be studied with careful control of experimental stimuli. Thus, the third aim of the present study is to examine how comprehenders interpret scalar implicature and ad hoc implicature produced by L1 and L2 speakers and investigate how linguistic complexity affects L2 processing.

## 2. Judgments toward L1 and L2 speech

Speaking with a foreign accent influences L2 speakers in two respects. First, a foreign accent requires additional processing efforts since it is an acoustic deviation from stored phonological representations in native language norms (Van Engen and Peelle, 2014). It further reduces the intelligibility of the speech and makes it difficult to understand (Munro and Derwing, 1995; Lev-Ari and Keysar, 2012). Moreover, the foreign accent serves as a signal to show that the L2 speaker is an out-group member with respect to the target language community since the other L1 speakers all speak the target language without an accent. This outsider-signal can generate prejudice against L2 speakers (Dixon et al., 2002). Due to these two influences, an identity as L2 speaker is often thought to convey disadvantages, such as being judged to be less intelligent and competent (Reisler, 1976; Kalin and Rayko, 1978), less trustworthy (Lev-Ari and Keysar, 2010), and less hirable (Huang et al., 2013). Unlike these findings indicating a negative social evaluation of L2 speakers, Fairchild and Papafragou (2018) and Fairchild et al. (2020) reported that being a foreign-accented L2 speaker could lead to certain advantages, as such individuals are more easily forgiven when they fail to provide informative statements.

In their study, Fairchild and Papafragou (2018) manipulated speaker identity (i.e., L1 speaker and L2 speaker) and investigated the interpretation of scalar implicature by English-speaking comprehenders: in the L1 speaker condition, utterances were made by Emma, a L1 English speaker with a strong Boston accent; while in the L2 speaker condition, utterances were made by Yuqi, a L1 speaker of Mandarin Chinese with a strong Chinese accent. To ensure that the intelligibility of the utterances remained constant while the speaker identity changed, the authors used written sentences as stimuli and told the participants clearly that these English sentences were uttered by a L1 speaker and a L2 speaker. They instructed participants to read these written sentences and then to complete a sentence rating task. In the first experiment, underinformative sentences (e.g., “Some dogs are mammals”) that violated the Gricean Quantity Maxim were used as target items. The results showed that participants interpreted the utterances of L1 and L2 speakers in systemically different ways even without a physical accent to process. That is, participants gave higher ratings to the underinformative sentences ascribed to the L2 speaker than those ascribed to the L1 speaker, thus indicating that the L2 speakers’ low L2 proficiency characterized by a foreign accent allowed participants to forgive their information-omitting behavior more easily. The results of Experiment 2 and Experiment 3 fully replicated this finding in the context of a new set of stimuli and demonstrated that participants tended to tolerate L2 speakers’ underinformative utterances. It is worth noting that in Experiment 2, a third speaker condition was added, i.e., a L2 speaker named Peiyao who was from China but had no Chinese accent whatsoever. The results showed that this accent-free L2 speaker was treated in a similar manner to a L1 speaker and so received no advantage with respect to participants’ leniency.

However, the explanation for these distinctively different attitudes toward L1 and L2 speakers’ utterances remains unknown. Unlike the judgment task used in Fairchild and Papafragou (2018), Fairchild et al. (2020) asked participants to write down possible reasons to explain why speakers made underinformative statements to investigate the ways in which underinformative sentences were interpreted and the ways in which L2 speakers’ identity functioned as a social benefit. The results of Experiment 1 and Experiment 2 in this study showed that participants explained L2 speakers’ underinformativeness as a result of inability while unwillingness was the explanation they provided for L1 speakers. Since unwillingness was often associated with misleading intentions and deception, it was more likely to be penalized. Therefore, participants’ choices demonstrated lenience toward L2 speakers. In this study, written sentences also served as stimuli, and speakers’ identity was manipulated by presenting participants with different sets of biographical information that indicated a L1 speaker and a L2 speaker.

These two studies have contributed to providing a more complete picture of L2 speech processing and social evaluation of L2 speakers. However, it should be noted that both of the studies used English as the target language, and utterances were judged by L1 speakers of English. The question of the extent to which the results of these studies can be generalized to different target languages and foreign accents remains intriguing but underexplored. Given that previous studies have already shown that comprehenders’ perception of accented speech is greatly impacted by their cultural backgrounds (Heald and Nusbaum, 2014), the current research aims to explore Chinese comprehenders’ judgment and understanding of foreign-accented discourse. Additionally, the current study is also interested in how comprehenders’ understanding of the message conveyed by a foreign accented speaker leads to differences in perception of the speaker. Therefore, in this study speakers’ perceived characteristics are assessed by asking participants to rate speakers on different attributes. According to theories in social cognition, the warmth dimension and the competence dimension are two universal dimensions of person perception. Honesty and reliability belong to the warmth dimension which reflects traits that are related to perceived intent; perspective taking and communication skills belong to the competence dimension which represents traits that are related to perceived ability (Fiske et al., 2007). In addition, the interrelationships between participants’ behavioral choices (i.e., their choice to view speakers as potential friends or colleagues), and their evaluation of accented utterances have also been highlighted by several studies (e.g., Trofimovich and Turuševa, 2020). Therefore, in line with the findings of social cognition and accent research we chose these five attributes (honesty, reliability, perspective-taking, likelihood of being friends and communication skills) to examine the effects of L2 accent on the social meaning of utterances.

## 3. Underinformative utterances and implicature in context

In communication, interlocutors are very often required to go beyond the literal meaning of others’ utterances to infer the intended and implied meaning. Following Grice (1975, 1989), interlocutors are expected to be cooperative and to obey a series of conversational maxims. For example, the Quantity Maxim requires speakers to make their contribution as informative as necessary (for the current purposes of the exchange) and not to make their contribution more informative than is needed. In other words, cooperative speakers are supposed to say no less and no more than what is needed for the purposes of the conversation. However, it is not the case that everything a speaker says or writes is genuinely as informative as the Quantity Maxim stipulates (Grice, 1989; Arts et al., 2011; Gatt et al., 2014). For instance, in saying (1), a speaker violates the maxim if the fact is all of the students passed the exam. Namely, interlocutors often identify the pragmatic meaning of (1) as indicating that “Some but not all students passed the exam,” and a more informative statement would be “All the students passed the exam.”

(1) Some students passed the exam.

Inferring “not all” from “some” is an example of scalar implicature from the scale <some, all> regarding informational strength ranging from weak to strong (Horn, 1972). When one interlocutor makes an underinformative statement by using a relatively weaker scalar item, others would wonder about the speaker’s reason for not using the stronger one. First, the fact that the speaker chooses some suggests that the speaker is not sufficiently well-informed to believe the stronger alternative all to be true possibly due to a lack of information (Horn, 1972; Grice, 1975; Carston, 1998; Sauerland, 2012; Goodman and Stuhlmüller, 2013). Additionally, this underinformativeness can also be attributed to the speaker’s intention to deceive others by omitting information. In such a case, speakers are underinformative due to their unwillingness to provide additional information.

Scalar implicature is regarded as a form of generalized (conversational) implicature in that the pragmatic derivation is usually part of a lexical scale. In contrast, ad hoc implicature is a form of particularized (conversational) implicature, such that the pragmatic inference is generated from a real-world context. Imagine that a context includes two bags, one of which contains a butter croissant while the other contains both a chocolate croissant and a butter croissant. If someone says that,

(2) The bag with a butter croissant is mine.

this statement implies that the bag with only a butter croissant is mine, not the bag with both a butter and a chocolate croissant. The context generates a contrast between the implicature and the alternative reading “The bag with a butter croissant and a chocolate croissant is mine.” If the alternative reading is what the speaker intends to express, sentence (2) is underinformative. It should be noted that although both types of implicature are closely related to the Quantity Maxim, alternative readings are derived in a distinctively different manner in each case: alternatives in scalar implicature are linguistically predetermined on a lexicalized scale, whereas alternatives in ad hoc implicature are determined by a particular situation in a specific context. The difference in linguistic difficulty between scalar and ad hoc implicature has been demonstrated by empirical data. It has been found that deriving scalar implicature is more difficult for children, and this difficulty might result from children’s lack of knowledge regarding scalar alternatives to words such as *some* (Papafragou and Musolino, 2003; Barner et al., 2011; Horowitz et al., 2017; Foppolo et al., 2021). Foppolo et al. (2021) used two picture selection tasks to compare children’s ability to derive scalar and ad hoc implicature, and the results showed that children derived ad hoc implicature in a manner similar to adults but did not derive scalar implicature in a comparable fashion, thus revealing a significant effect of the type of implicature on children’s performance. Studies of Chinese speakers’ ability to compute scalar and ad hoc implicature have reported similar results. Zhao et al. (2021) made direct comparisons between Mandarin-speaking children’s computations of scalar and ad hoc implicature. The findings indicated that scalar implicature was difficult for Chinese children, and consistent success in deriving such implicature were not observed until the age of six, while children were able to compute ad hoc implicature by the age of four. However, in the works of Fairchild and colleagues, the effect of the type of implicature in question has received little attention. In Fairchild et al. (2020) participants’ justifications for statements containing ad hoc implicature were used to explain the leniency observed with respect to utterances containing scalar implicature in Fairchild and Papafragou (2018). Nevertheless, participants’ leniency and their justification for that leniency might be influenced by linguistic difficulties in deriving different types of implicature. Therefore, in the current study, by comparing participants’ comprehension of scalar and ad hoc implicature, we aim to determine whether linguistic difficulties in the derivation of different types of implicature influence their evaluations of underinformativeness and their social judgments of speakers.

## 4. Research questions

The study seeks to address the following research questions:

1.Do L1 Chinese comprehenders comprehend underinformative utterances made by L2 speakers differently from those made by L1 speakers? In other words, are L2 speakers more likely to be tolerated than L1 speakers when they produce underinformative sentences?
2. Does the difference in linguistic complexity between scalar and ad hoc implicature influence the tendency of Chinese comprehenders to tolerate L2 speakers’ failures to produce informative statements?
3.Do explanations of underinformative utterances differ in accordance with the identity of the speaker?
4.Do speakers’ perceived characteristics differ in accordance with the identity of the speaker?

First, Chinese comprehenders were expected to show leniency with respect to underinformative sentences produced by L2 speakers, as in the research by Fairchild and Papafragou (2018). Second, the more linguistically complex nature of scalar implicature should result in greater leniency of information omission than that associated with ad hoc implicature. Additionally, inability was predicted to be used to justify L2 speakers’ underinformative utterances more frequently than the underinformative utterances of L1 speakers. L1 speakers’ omission of information was more likely to be associated with unwillingness. Since the strength of L2 speakers’ accent is often interpreted as a marker of their second language proficiency (Kang et al., 2010), the accented L2 speaker may receive higher leniency than the accent-free L2 speaker. Lastly, given that previous studies showed that L2 speakers are often associated with negative biases, L2 speakers were predicted to receive low ratings on different characteristics.

## 5. Materials and methods

### 5.1. Test design

This experiment adopted a sentence judgment task to investigate the ways in which underinformative statements including scalar and ad hoc implicature are processed when ascribed to L1 and L2 speakers. Two factors were manipulated in a 3 × 2 design: speaker identity type and implicature type. The speaker identity type included three levels, namely, L1 speaker Tianqi, accent-free L2 speaker Emma and foreign accented L2 speaker John. With the aim of priming participants with expectations about the speaker, the current experiment used guises, i.e., different biographical information of 3 speakers and an image that aligned with their identity to meet participants’ expectations.^2^ The implicature type factor included two levels, namely, ad hoc implicature and scalar implicature. Additionally, when designing the underinformative contexts of scalar implicature, we also took question under discussion (QUD) into consideration since the likelihood of deriving scalar implicature is largely dependent on the relevance under a specific QUD (Roberts, 2004, 2012). The results of relevant studies have shown that QUD affects the interpretation of scalar items such as *some* (Zondervan, 2009; Yang et al., 2018). It has been found that participants were more likely to provide higher ratings to the underinformative sentences in the lower-bound QUD (i.e., containing the weaker scalar item *any*) than in the upper-bound QUD (i.e., containing the stronger scalar item *all*). In other words, the upper-bound QUD triggers the scalar implicature more often than the lower-bound QUD. Since the conversation in our test items involved a question and an answer, we used both upper-bound QUD and lower-bound QUD (see Table 1) to balance the possibility of triggering scalar implicature.^3^

**Table 1:** Examples of target underinformative stimuli of ad hoc and scalar implicature.

	Ad hoc implicature	Scalar implicature
Context	There is a turkey and an apple in the basket.	There are five apples in the basket.
Cashier’ question	*What’s in the basket?* (篮子里有什么?)	*Are all the things in the basket apples?* (upper-bound QUD) (篮子里全都是苹果吗?) *Are there any apples in the basket?* (lower-bound QUD) (篮子里有苹果吗?)
Character’s answer (underinformative)	*In the basket, there is an apple.* (篮子里有一个苹果。)	*In the basket, some are apples*. (篮子里有些是苹果。)

**Figure 1:**
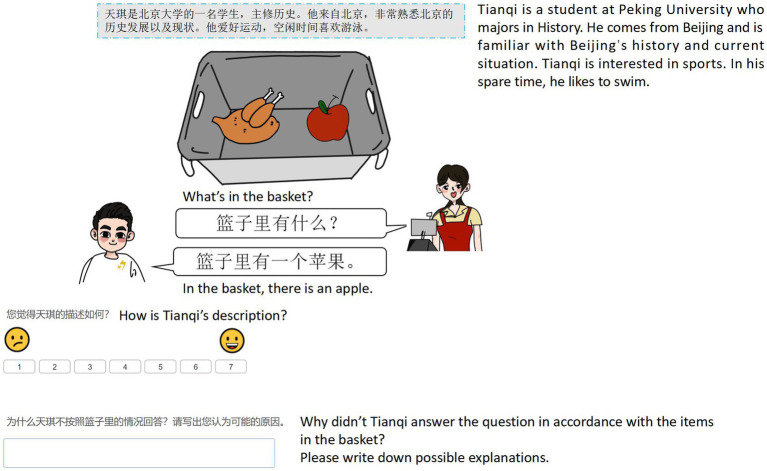
Sample display of an underinformative item in ad hoc implicature (Speaker type: L1 speaker).

**Figure 2:**
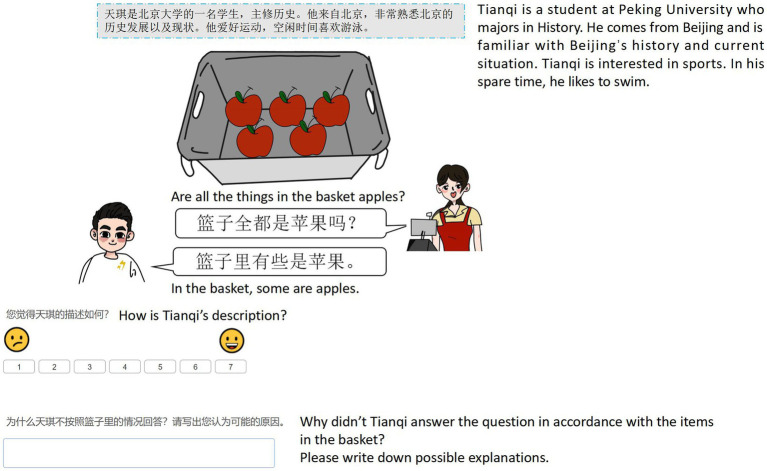
Sample display of an underinformative item in scalar implicature (Speaker type: L1 speaker).

Participants, first of all, read biographical information regarding three characters, and they were told that these three characters were in the process of checking out at a supermarket. Specifically, the characters were answering a question from a cashier by describing what he or she had inside his or her shopping basket (see Figures 1,2 for example displays). Participants were then asked to read the speaker’s description and rate it on a 7-point Likert scale. The last trial for each speaker condition (including fillers) also included an open-ended question asking participants to provide justifications for the speaker’s utterance.^4^ It was intended to explore whether participants attributed speakers’ intentions in using underinformative statements to various reasons based on the speaker’s identity. Participants’ responses to this question were later coded following the common practice discussed in Miles and Huberman (1994). Three rounds of coding were conducted to ensure validity and reliability. First, two researchers categorized the same 10% of responses that were randomly selected from the dataset and then reviewed each categorization together. They clarified and discussed differences until intercoder reliability reached 100%. The same process was repeated again for another 10% of responses. Finally, each researcher finished categorizing and reviewing the rest of the responses. Eventually, both intra-and intercoder agreement rates reached 94%, and consensus was made regarding the remaining differences after discussion.

Figures 1, 2 are two sample displays of ad hoc and scalar implicature for the target underinformative condition with the L1 speaker. Two types of fillers with similar structures were also included. Logically false statements should be rejected (false condition), e.g., in scalar implicature, the description “In the basket, some are watermelons” is false when presented with a basket with five bananas. The other type is pragmatically and logically felicitous sentences that should be accepted (optimal condition). For example, in ad hoc implicature, the description “In the basket, there is a pumpkin and an orange” is optimal when presented alongside a basket containing a pumpkin and an orange.

Eighteen target underinformative items (12 items for scalar implicature with two QUDs and 6 items for ad hoc implicature) and 24 filler items were constructed for each speaker. Thus, for all the three speakers, 54 (18 * 3) target items and 72 (24 * 3) filler items were available for each participant to read. After all test stimuli had been finalized, two presentation lists were created to counterbalance the presentation order of the two L2 speakers, namely, List A (order: Tianqi, Emma, John) and List B (order: Tianqi, John, Emma). Two lists were presented to two different groups of participants.

Written sentences were used as stimuli. The use of physical voices or audio recordings of actual foreign accents was not chosen since we needed to ensure that utterance intelligibility was identical throughout the experiment even though sentences were attributed to different types of speakers. Written stimuli also eliminated the effect of different processing demands (Fairchild and Papafragou, 2018; Fairchild et al., 2020).

### 5.2. Participants and procedures

Eighty-one L1 Chinese speakers participated in this study and were randomly assigned to one of the two groups in accordance with the presentation list: List A (n = 41) and List B (n = 40). The ages of participants ranged between 18 and 36, and participants included 30 men and 51 women. Participants were asked to report their English-language proficiency. 85% participants had passed CET-4 (College English Test Band 4), a test conducted by the National College English Testing Committee to examine the English proficiency of undergraduate students in China. The rest of participants reported their test scores in the National College Entrance Examination. Participants were compensated for their participation.

The experiment was administered online *via* Credamo^5^, a professional data platform similar to Qualtrics Online Sample that provides large-scale collection services. Prior to the beginning of the experiment, participants were asked to read and sign an electronic consent form. Subsequently, participants read biographical information concerning each of the three speakers (see Table 2) one by one.

**Table 2:** Speaker bios.

L1 speaker	Accent-free L2 speaker	Accented L2 speaker
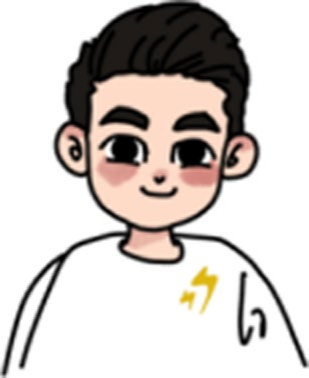 **Tianqi** is a student at Peking University who majors in History. He comes from Beijing and is familiar with Beijing’s history and current situation. Tianqi is interested in sports. In his spare time, he likes to swim.	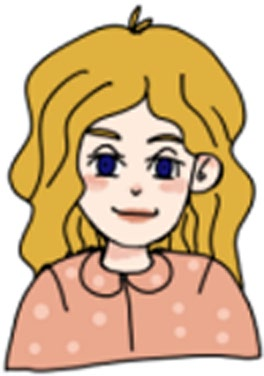 **Emma** is an international student at Peking University who majors in Art. She comes from America. She speaks Chinese with no American accent and people often mistakes her for being Chinese. Emma is interested in food. In her spare time, she likes to cook.	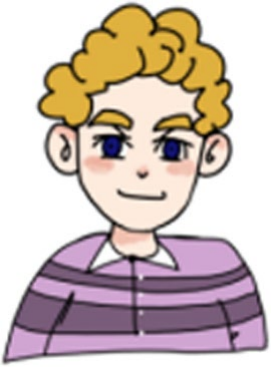 **John** is an international student at Peking University who majors in Sociology. He comes from America. He speaks Chinese with a strong American accent and he sometimes needs to repeat a sentence several times before others can understand. John is interested in music. In his spare time, he likes to play the piano.

After reading written instructions of the task, the experiment started. Depending on the presentation list (A or B), the order of the two L2 speakers varied across the two groups; however, the task procedures for each speaker were identical. That is, the speakers’ biographical information was presented to the participants once again; subsequently, participants answered two comprehension questions related to information in the speaker biography to ensure that they had read the biographical information carefully; then, participants completed 42 trials (18 targets and 24 fillers) that were operationalized as shown in Figures 1, 2; finally, participants were asked to rate five attributes related to the speaker on a 4-point Likert scale, namely, honesty, reliability, perspective-taking, likelihood of being friends and communication skills. Participants repeated the procedures discussed above for the other two speakers. Note that speakers’ demographic information was always visible in a box at the middle of the screen to remind participants of this information. At the conclusion of the entire experiment, participants were required to complete a demographic survey to provide information concerning their age, gender, and English proficiency scores. All the information provided in the experiment, including the instructions, speaker biographies, test stimuli and participants’ responses, was in Chinese. The overall experiment took approximately 20 min to complete.

## 6. Results

Prior to analysis, the data were trimmed by verifying the accuracy of participants’ answers to the comprehension questions regarding the speakers’ biographies. Participants’ data were removed if they selected an incorrect answer more than twice. This process resulted in the removal of data of only one participant who participated in List A. Participants’ justifications were coded into four classes, namely, inability, unwillingness, metalinguistic awareness and L1 influence on way of thinking. In the following discussion of the results, we first present ratings of utterances on the 7-point scale (with a focus on the target underinformative condition) to answer the first two research questions. Subsequently, we focus on Chinese comprehenders’ justification of underinformative utterances based on the four classes for the third question. Finally, to answer the fourth question, ratings of speakers’ attributes on the 4-point scale are discussed.

### 6.1. Acceptability judgment data

**Figure 3:**
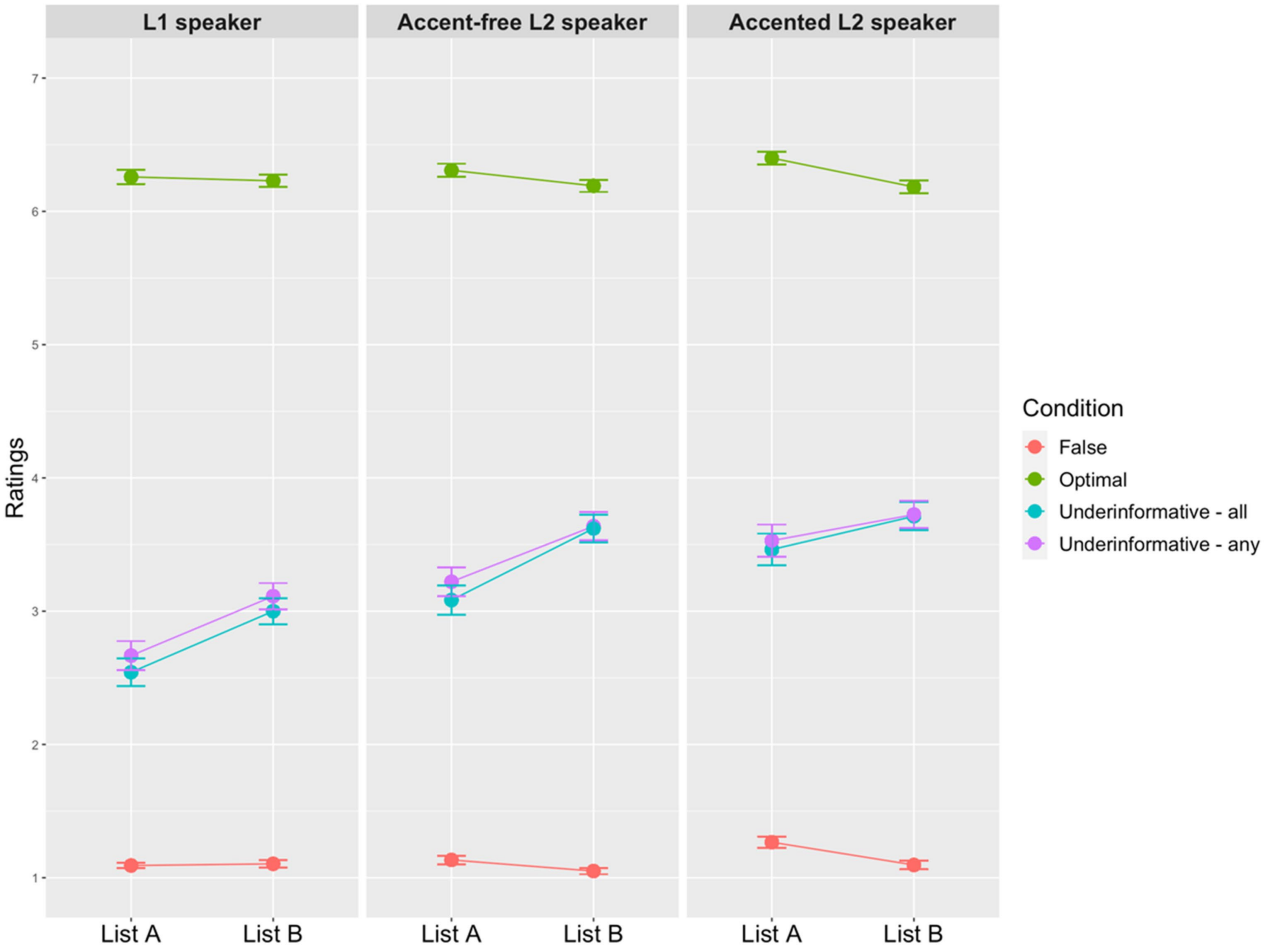
Mean ratings of all conditions for scalar implicature with points and standard error bars (the *y*-axis represents the acceptability of sentences. The higher the ratings are, the more acceptable the sentences are).

**Figure 4:**
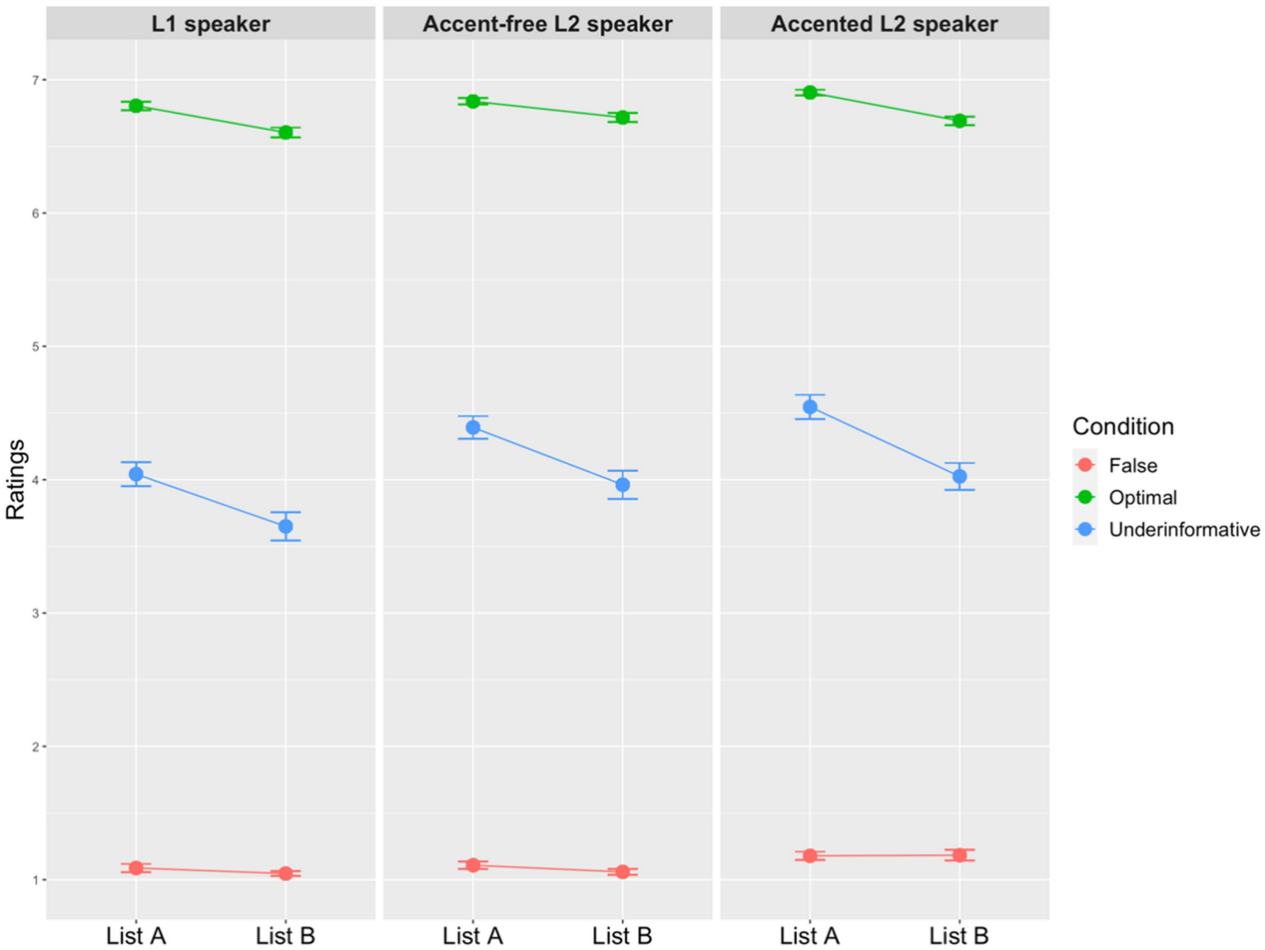
Mean ratings of all conditions for ad hoc implicature with points and standard error bars (the *y*-axis represents the acceptability of sentences. The higher the ratings are, the more acceptable the sentences are).

Figures 3, 4 show the mean ratings of scalar and ad hoc implicature depending on the presentation order. Generally speaking, ratings of both scalar and ad hoc implicature were similar in the overall response pattern, i.e., underinformative conditions were rated lower than the optimal condition but higher than the false condition. To compare ratings across all the conditions in each type of the implicature, two separated cumulative link mixed effect models (CLMM) were run by using the clmm() function from the ordinal package in R (Christensen, 2015). The dependent variable was the 7-point Likert scale ratings. The fixed effects within each model were as follows: Condition (scalar implicature: 4 levels, optimal, underinformative-“all,” underinformative-“any,” false; ad hoc implicature: 3 levels, optimal, underinformative, false); Speaker (3 levels: L1 speaker, accent-free L2 speaker, accented L2 speaker); and List (2 levels: List A-Tianqi-Emma-John, List B-Tianqi-John-Emma). We used dummy contrast coding for the fixed effects of Condition (baseline level for scalar implicature: underinformative-“any”; baseline level for ad hoc implicature: underinformative), Speaker (baseline level: L1 speaker) and List (baseline level: A). Unless otherwise mentioned, we followed the common practice suggested by Barr et al. (2013) for maximal random effects structure. However, since the maximal model did not converge, the random effect structure was simplified following the back-off procedure (Bates et al., 2015). The final models that converged for the two models included by-item varying intercepts, as well as by-participant varying intercepts and varying Speaker slopes.

To answer the first research question, regarding scalar implicature, the model output (Supplementary Table S1) shows that there was a significant interaction between Condition and List, as well as a significant interaction between Condition and Speaker. The three-way interaction was not significant. The two significant interactions were further analyzed in pairwise comparison tests. The results indicated that all the four conditions were rated similarly between List A and B (all *p*s > 0.1). Interestingly, for both lists, participants’ ratings did not differ between the two QUDs (List A: *Z* = 1.850, *p* = 0.586; List B: *Z* = 0.665, *p* = 0.998). That is, participants provided similar ratings regardless of different QUDs. Additionally, this insensitivity to QUD did not differ within each Speaker type (all *p*s > 0.1). The pairwise comparison also found that in the optimal and false conditions, the ratings among the three Speaker type did not differ significantly from each other (all *p*s > 0.1). What’s important was in the two underinformative conditions, the L1 speaker was rated significantly lower than the two L2 speakers (all *p*s < 0.0001) while ratings between the two L2 speakers were similar (any: *Z* = −2.435, *p* = 0.382; all: *Z* = 2.928, *p* = 0.131). It suggested that participants gave significantly higher ratings to the two L2 speakers than the L1 speaker in two underinformative conditions.

The model including all the conditions in ad hoc implicature (Supplementary Table S2) indicated effects of Condition and Speaker. The underinformative condition was rated significantly lower than the optimal condition but higher than the false condition. The L1 speaker was also rated significantly lower than the two L2 speakers. More importantly, ratings in the two lists were similar.

Next, we take a specific look at participants’ ratings of underinformative utterances in the two types of inference (the second research question). Results in the previous analysis of scalar and ad hoc implicature (i.e., Supplementary Tables S1, S2) indicated that ratings did not differ significantly between the two lists, and therefore, in this analysis, we combined ratings in the two lists together for each type of implicature. The *clmm* model included Type (2 levels: scalar and ad hoc implicature) and Speaker (3 levels: L1 speaker, accent-free L2 speaker, accented L2 speaker) as the fixed effects. The random effects were by-participant varying intercepts and varying slopes for Speaker; by-item varying intercepts and varying slopes for Speaker. The model output (Supplementary Table S3) showed a significant interaction between Type and Speaker. Pairwise comparisons revealed that within the three speakers, underinformative utterances in ad hoc implicature were rated significantly higher than the ones in scalar implicature (all *p*s < 0.0001), while speakers’ underinformative utterances were treated differently depending on the type of inference. For instance, the ratings of underinformative statements by the accented L2 speaker in ad hoc implicature was rated significantly higher than the ones by the L1 speaker (*Z* = −2.911, *p* = 0.04). Additionally, in scalar implicature, the L1 speaker received much lower ratings than the two L2 speakers (L1 speaker vs. accent-free L2 speaker: *Z* = −5.308, *p* < 0.0001; L1 speaker vs. accented L2 speaker: *Z* = −6.282, *p* < 0.0001), whereas there was no rating difference between the two L2 speakers (*Z* = −2.279, *p* = 0.21).

### 6.2. Justification data

Participants’ justifications of ratings were coded in order to answer our third research question. Four categorizations were found as shown in Table 3, including inability and unwillingness as the two major classes and two other classes. The two major classes followed the categorization employed in Fairchild et al. (2020) with minor differences concerning the subcategories of unwillingness. We excluded “politeness” and “saving face” in Fairchild and Papafragou (2018) since no related responses were found; two new subcategories related to “avoidance” were added for situations in which participants explained speakers’ unwillingness in terms of avoidance. The avoidance explanations were categorized into “linguistic avoidance” (e.g., “He thought he had an accent when saying XX so he avoided saying that word”) and “emotional avoidance” (e.g., “He does not want to look too radical”). Although both groups of speakers were mostly associated with “emotional avoidance,” L2 speakers were also associated with “linguistic avoidance.” Responses that indicated participants’ awareness of the upper-bound and lower-bound QUDs and logical readings of implicature were categorized as metalinguistic awareness (e.g., “Some can logically mean all”). The final categorization, L1 influence on way of thinking, included responses indicating differences in way of thinking between Chinese and English (e.g., “The speaker follows his/her English way of thinking”).^6^

**Table 3:** Justifications given for underinformative utterances as categorized by speaker type and justification type.

Speaker and Implicature typeJustification	L1 speaker		Accent-free L2 speaker		Accented L2 speaker	
	Ad hoc	SI	Ad hoc	SI	Ad hoc	SI
Inability	42.65%	55.28%	55.41%	63.36%	76.32%	76.60%
-Linguistic difficulty	5.88%	4.88%	13.51%	29.77%	34.21%	48.23%
-Perceptual or cognitive difficulty	36.76%	50.41%	41.89%	33.59%	42.11%	28.37%
Unwillingness	48.53%	35.77%	36.49%	23.66%	19.74%	12.05%
-Deception	22.06%	16.26%	14.86%	7.63%	6.58%	4.96%
-Linguistic avoidance	0%	0%	4.22%	3.82%	5.26%	3.54%
-Emotional avoidance	26.47%	19.51%	17.4%	12.21%	7.9%	3.55%
Metalinguistic awareness	8.82%	8.95%	4.05%	5.34%	0.00%	2.13%
L1 influence on way of thinking	0.00%	0.00%	4.05%	7.63%	3.94%	9.22%

Regarding inability, L1 speaker’ s underinformative utterances were mostly related to perceptual or cognitive difficulties regarding the two types of implicature (e.g., ad hoc implicature: “He did not remember that he bought the apple,” “He did not see the apple”; scalar implicature: “He did not remember how many apples he bought,” “He did not see all the apples”). The utterances of the accent-free L2 speaker were also mostly associated with perceptual or cognitive difficulties for the two types of implicature; however, proportion tests showed that the accent-free L2 speaker’ rate of linguistic difficulty was much higher than the L1 speaker for scalar implicature (*χ*^2^ = 26.453, *p* < 0.0001), but not for ad hoc implicature (*χ*^2^ = 1.754, *p* = 0.185). The justifications of the accented L2 speaker differed from those of the accent-free L2 speaker in that for scalar implicature, linguistic difficulty was the dominant class of justifications used for the accented L2 speaker (approximately 48%).

With respect to unwillingness, the L1 speaker received the highest percentage of responses related to unwillingness (especially 48.53% in ad hoc implicature), and the accented L2 speaker received the lowest percentage (approximately 12% to 19%). A frequent deception and avoidance explanation for the L1 speaker was “He was lying” and “He does not want to be too radical” respectively. Regarding deception, the accent-free L2 speaker resembled the L1 speaker in the case of ad hoc implicature (*χ*^2^ = 0.600, *p* = 0.439); however, the accented L2 speaker was less likely associated with deception than native speakers (*χ*^2^ = 5.955, *p* = 0.0147). Interestingly, there was a high percentage of metalinguistic awareness for the L1 speaker and accent-free L2 speaker. Finally, L1 influence on way of thinking was only present for the two L2 speakers and it was more frequently associated with scalar implicature.

### 6.3. Attribute data

**Figure 5:**
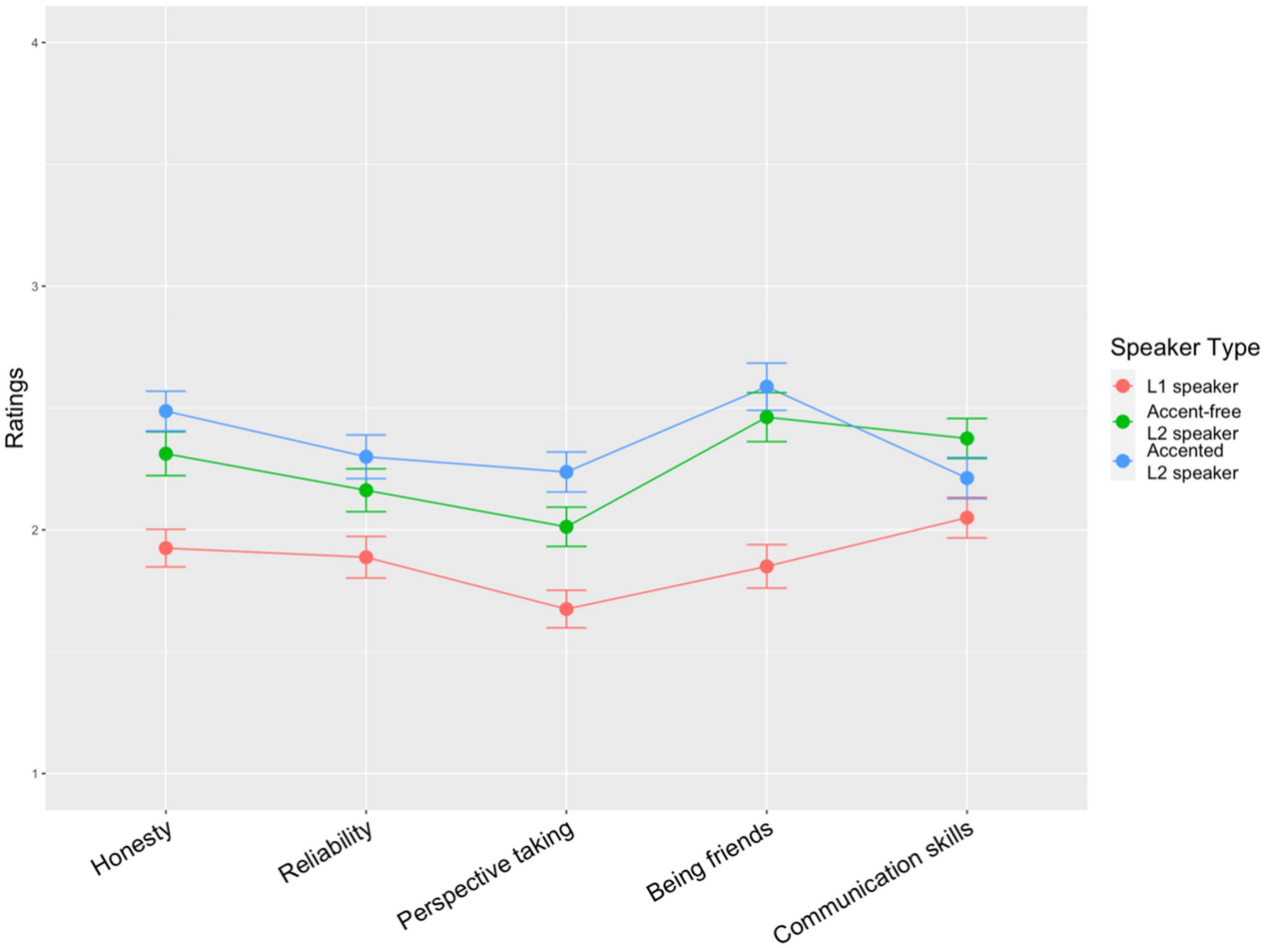
Mean ratings of the five attributes with points and standard error bars (the *y*-axis represents the degree of each attribute, e.g., the higher the ratings of honesty are, the more honest the participants think the speaker is).

The final step in this task was to ask participants to rate the speaker’s attributes, namely honesty, reliability, perspective-taking, likelihood of being friends and communication skills on a 4-point scale (the fourth research question). Figure 5 shows that the L1 speaker was rated much lower than the two L2 speakers regardless of different attributes. For all the three speakers, the lowest attribute was perspective-taking. The accented L2 speaker received slightly higher ratings than the accent-free L2 speaker in all the five attributes except communication skills. A *clmm* model was fitted to the data using the 4-point scale ratings as the dependent variable and Speaker (3 levels: L1 speaker, accent-free L2 speaker, accented L2 speaker) and Attribute (5 levels: honesty, reliability, perspective-taking, likelihood of being friends and communication skills) as the fixed effects. The random effects were by-participant varying intercepts and varying slopes for Speaker; by-item varying intercepts and varying slopes for Speaker. The model output (Supplementary Table S4) revealed a significant interaction between Speaker and Attribute. Pairwise comparisons suggested that, first, the two L2 speakers did not differ from each other in all the five attributes and the three speakers received similar ratings in communication skills (all *p*s > 0.1). Secondly, the L1 speaker was rated significantly lower than the accented L2 speaker in honesty (*Z* = −4.732, *p* < 0.001), reliability (*Z* = −3.648, *p* = 0.022), perspective-taking (*Z* = −4.927, *p* < 0.0001) and likelihood of becoming friends (*Z* = −4.898, *p* < 0.0001). The L1 speaker also received marginally significant lower ratings than the accent-free L2 speaker in honesty (*Z* = −3.251, *p* = 0.07). In addition, the L1 speaker received significantly lower ratings than the accent-free L2 speaker in the likelihood of becoming friends (*Z* = −4.169, *p* = 0.003).

## 7. Discussion

While some studies suggested that L2 speakers are often the subject of negative biases (Lev-Ari and Keysar, 2010; Hanulíková et al., 2012; Gibson et al., 2017; Grey and van Hell, 2017), Fairchild et al. (2020) and Fairchild and Papafragou (2018) found that English comprehenders processed L1 and L2 speakers’ underinformative utterances differently, and this difference extended to L2 speakers a positive bias with respect to judgments. We extended this line of research to Chinese comprehenders who have a different cultural and linguistic background than participants in previous studies and included two types of implicature, namely scalar implicature and ad hoc implicature. Overall, the findings of our study clearly indicated that speaker identity plays an important role in Chinese comprehenders’ assessment of underinformative sentences and that foreign accents confer on L2 speakers the social benefit of being tolerated for their omission of information in communication and that of being viewed as more likely to possess positive attributes.

### 7.1. Benefits of foreign accent in L2 language processing

One of the aims of this study was to determine whether Chinese comprehenders understand underinformative utterances made by L2 speakers with or without foreign accent differently from the way in which they understand those of L1 speakers. Crucially, the results of the current study replicated the main findings of Fairchild and Papafragou (2018) and Fairchild et al. (2020), who reported that speaker identity plays an important role in comprehenders’ assessment of underinformative sentences made by L1 and L2 speakers. Acceptability ratings of both scalar and ad hoc implicature in the current study clearly indicated that Chinese comprehenders rated underinformative utterances made by L2 speakers higher than those made by the L1 speaker, thus suggesting that the comprehenders were more lenient and tolerant of pragmatic violations made by L2 speakers. Underinformative utterances by L2 speakers were more likely to be attributed to inability rather than unwillingness. More importantly, the ratio of inability justification increased as the Chinese proficiency decreased. The strength of L2 speakers’ accent is often interpreted as a marker of their second language proficiency (Kang et al., 2010). The accented L2 speaker received the highest percentage of inability justification (approximately 76%), even higher than the accent-free L2 speaker (approximately 55% in the ad hoc condition and 63% in the SI condition), regardless of the fact that they are both L2 speaker. It suggests that Chinese comprehenders were not only tolerant of pragmatic violations made by L2 speakers, but also were sensitive to L2 speakers’ estimated language proficiency indexed by the strength of accent. In addition, difficulties with remembering and seeing was the dominant subcategory in inability for the L1 speaker, whereas L2 speakers were more often associated with linguistic difficulties. Explanations related to unwillingness, whether in terms of deception or in terms of avoidance, were associated less frequently with L2 speakers than with the L1 speaker. In the attribute rating task, the L1 speaker was also rated to be less honest than the two L2 speakers. The findings of the current study demonstrated that social benefits suggested by higher tolerance and leniency of underinformative utterances were more often provided to L2 speakers than the L1 speaker.

Moreover, with respect to the relationship between the strength of the speaker’s foreign accent and the speaker’s likelihood of receiving social benefits, another evidence we have found is that the accented L2 speaker is being viewed as more likely to possess positive attributes: the accented L2 speaker received significantly higher ratings in honesty, reliability, perspective taking and likelihood of becoming friends than the L1 speaker. A growing body of literature in accent research has reported evidence concerning the effect of accent on personal attributes, especially in the fields of social psychology and organizational science. Studies have found that people with heavy accents are judged to be less honest and reliable (e.g., Lindemann, 2003; Lev-Ari and Keysar, 2010). To some researchers, L2 accent signals poor communication skills (e.g., Gluszek and Dovidio, 2010). In addition, L2 accented speakers were rated less favorably on friendly traits and this further affected participants’ behavioral choices (e.g., Giles and Watson, 2013). Findings of the current research are inconsistent with previous research and we propose a preliminary explanation for the variation of different attributes the three speakers received with reference to theories and evidence in social cognition. As discussed above, participants in the current study gave more “unwillingness” justifications, especially “deception” to the L1 speaker for his underinformative utterances, a category which is attributed with ill intent. In contrast, the category “inability” is the major justification given to the two L2 speakers, a category which is perceived as less negative than “unwillingness.” According to the recent theory and research in social cognition, warmth and competence are two universal dimensions of social cognition. Traits in the warmth dimension (i.e., honesty and reliability in the current study) are primary and carry more weight than traits in the competence dimension (i.e., perspective and communication skill in the current study) in people’s behavioral reactions (Peeters, 2002; Fiske et al., 2007). Since honesty and reliability are two traits relating to speakers’ perceived intent and the L1 speaker is regarded as giving underinformative utterances with ill intent, it is reasonable that the L1 speaker received low ratings in these two attributes. Ratings in the likelihood of becoming friends demonstrate participants’ behavioral preferences based on their judgments about speakers’ traits in both warmth dimension and competence dimension. Therefore, participants might be more likely to rely on their judgments about speakers’ honesty and reliability when they were deciding whether they would make friends with speakers. In other words, significantly lower ratings received by the L1 speaker in honesty and reliability resulted in significantly lower ratings in the likelihood of becoming friends. In addition, the three speakers received similar ratings in communication skills. This may be explained by the fact that three speakers all provided underinformative responses to the cashier’s question and failed to facilitate the cashier’s work.

Although acceptability results were mostly in line with the conclusions of and Fairchild and colleagues, the justification results reported in our study revealed three new categories related to second/foreign language learning experiences that were absent in the work of Fairchild and colleagues. The first is the two categories related to avoidance. In explaining L2 speakers’ avoidance, there were some examples of linguistic avoidance, such as “He thought he had an accent when saying XX so he avoided saying that word.” These explanations suggested that participants were aware that an accent is normally associated with stigma and that they themselves might have experienced the intention of avoiding the stigma associated with uttering incomprehensible sentences in a second or foreign language. It should be noted that participants in the current study were all bilingual and had English as their second or foreign language.^7^ Presumably they have had experience in learning English under the influence of their L1—Chinese, e.g., speaking English with some Chinese accent (at least as a beginning learner). This experience of being a L2 speaker shares much more similarities with L2 speakers compared with monolingual speakers in Fairchild et al. (2020) and reduces participants’ accent bias since previous research has shown that first-hand experiences can change one’s attitudes and behavior (Brookhuis et al., 2011). As a result, it is very likely that these Chinese comprehenders projected their experience of learning a second or foreign language onto the L2 speakers described in the current experiment and further exhibit understanding toward the behavior of L2 speakers. The other new category is L1 influence on way of thinking for L2 speakers which took into account potential influence from the native language on way of thinking. The bilingual participants might have experienced uttering nontarget-like utterances under the influence of their native language. We would like to note that Chinese comprehenders’ likelihood of projecting their own language-learning experience onto others might also be affected by their East Asian cultural background since some research has found that East Asians attach importance to one’s relationship with others than westerners (Markus and Kitayama, 1991; Wu and Keysar, 2007). However, the influence of comprehenders’ bilingual experience and cultural background cannot clearly be isolated in the current experiment and therefore, future studies should tease apart the two factors in exploring how they affect comprehenders’ interpretation of underinformative statements in L1 and L2 speech.

### 7.2. The pragmatics of scalar and ad hoc implicature in L2 speech narratives

Unlike Fairchild and Papafragou (2018), Experiment 2, who reported that participants treated the accent-free L2 speaker as equivalent to the L1 speaker in terms of comprehending underinformativeness in scalar implicature, our results showed that the same effect was only present in ad hoc implicature. In interpreting scalar implicature, participants rated underinformative utterances made by the L1 speaker significantly lower than the two L2 speakers. In other words, Chinese comprehenders altered their degree of leniency with respect to interpreting different types of implicature based on their beliefs regarding the individual’s language proficiency, i.e., the two L2 speakers received greater the degree of leniency than the L1 speaker in scalar implicature, whereas the accent-free L2 speaker was treated as the L1 speaker in ad hoc implicature due to the high expectations of advanced second language proficiency suggested by their lack of a foreign accent. This suggests that type of implicature influences Chinese participants’ degree of leniency for underinformativeness. Next, we would like to discuss this result with respect to the fact that deriving scalar implicature is more complex than deriving ad hoc implicature. In addition to retrieving alternatives by reference to relevant contextual information, deriving scalar implicature also requires *a priori* knowledge of the alternatives that are lexicalized on the same scale <some, all>. Ad hoc implicature is relatively easier to derive since the context itself can provide sufficient resources for comprehenders to activate the alternatives. Thus, the knowledge of lexical scalar words on the scale of <some, all> poses an additional level of difficulty to L2 speakers. Scholars have argued that this difficulty is one of the reasons why children are less likely than adults to derive scalar implicature (Guasti et al., 2005; Barner et al., 2011; Foppolo et al., 2021). In our experiment, although the two L2 speakers were adults who had mature cognitive and pragmatic abilities and might be able to associate the two scalar items on the same scale in Chinese, the justifications associated with linguistic difficulty were still frequently mentioned in scalar implicature (29.77 and 48.23% for the two L2 speakers), more so than ad hoc implicature. And the accented L2 speaker, who had noticeably low proficiency suggested by the heavy accent, was attributed to linguistic difficulty at the rate of approximately 50%. Examples of justification of this category were related to L2 speakers’ failure to fully understand and use *some* and *all* in Chinese, e.g., “He has not fully acquired *youxie* (the Chinese counterpart of *some*) and *quanbu* in Chinese (the Chinese counterpart of *all*).” These justifications demonstrated that Chinese participants realized that scalar implicature involves *a priori* knowledge of the alternatives (similar justifications cannot be found with respect to ad hoc implicature^8^) and might be challenging to L2 speakers. Overall, in the current study, we find that the difficulty of the linguistic phenomenon affects the level of social benefits that L2 speakers receive. That is, if the linguistic construction is relatively easy, participants exhibit less tolerance of nontarget behavior (the accent-free L2 speaker can even be treated as L1 speaker, but only in ad hoc implicature).

An unexpected finding from the current study is that participants were not sensitive to the two QUDs, contrasting with previous studies (e.g., Dupuy et al., 2016). This does not necessarily mean that QUD does not have an influence on participants’ derivation of scalar implicature. The results merely suggested that in the current study QUD may not be the sole factor that participants take into account in their judgments of sentences with *some*. It is worth noting that the judgment tasks used in previous research were conducted based on picture-sentence matching tasks without a specific communicational purpose. Participants normally made a judgment based on whether the sentence matched the picture shown to them while no broader context was provided. The sentence judgment task in the current experiment, however, is different in that sentences were in an everyday language context in which the target sentence was a response to a cashier who needed to be informed the precise number of things in the basket in order to complete the checking-out process. In such case, only informative sentences with precise description of the basket fulfill the communication purpose and thus would be preferred and accepted. Therefore, even if participants are sensitive to QUD and interpret *some* under the lower-bound QUD as “some and possibly all,” this interpretation is still ambiguous in this context and leads to low ratings.

## 8. Conclusion

This study aims to examine the ways in which Mandarin Chinese speakers interpret underinformative utterances in L1 and L2 speech narratives. The results suggest that speaker identity affects Chinese comprehenders’ assessment of underinformative sentences and that foreign accents confer on L2 speakers the social benefit of being tolerated for their omission of information in communication and that of being more likely to be viewed as possessing positive attributes. Additionally, the linguistic difficulty of deriving scalar or ad hoc implicature affects the degree of social benefits that L2 speakers receive. Future research would benefit from exploring how cultural background and monolingual/bilingual experience influence comprehenders’ judgments of underinformative utterances. For instance, monolingual Chinese participants or bilingual Western participants should be enrolled as control groups in future studies, and it would be enlightening to discuss the ways in which these individuals interpret underinformative utterances made by L1 and L2 speakers.

## Data availability statement

The raw data supporting the conclusions of this article will be made available by the authors, without undue reservation.

## Ethics statement

Ethical review and approval was not required for the study on human participants in accordance with the local legislation and institutional requirements. The patients/participants provided their written informed consent to participate in this study.

## Author contributions

YL and SF contributed to conception and design of the experiment. YL implemented the experiment and SF oversaw the data collection. Both authors handled analysis of the experiments and creation of the manuscript.

## Funding

This research received support from The National Social Science Fund of China (# 20CYY002).

## Conflict of interest

The authors declare that the research was conducted in the absence of any commercial or financial relationships that could be construed as a potential conflict of interest.

## Publisher’s note

All claims expressed in this article are solely those of the authors and do not necessarily represent those of their affiliated organizations, or those of the publisher, the editors and the reviewers. Any product that may be evaluated in this article, or claim that may be made by its manufacturer, is not guaranteed or endorsed by the publisher.

## Footnotes

^1^We thank a reviewer for bringing this issue into our attention.

^2^For example, the two L2 speakers are shown in American faces in the visual display which are congruent with their biographical information (see Table 2). It should be noted that expectation of speakers can be affected by a variety of factors, such as race, language, nationality, evidenced by many empirical studies that have used guises in the experimental design (e.g., Babel and Russell, 2015; McGowan, 2015; Hansen et al., 2018; Vaughn, 2019). For example, McGowan (2015) discovered that when listening to Chinese-accented speech, listeners who were presented with a Chinese face were more accurate in transcribing than listeners who were presented with a Caucasian face. However, Hansen et al. (2018) showed that whether appearance or accent was presented first mattered. The relation among these factors is rather complicated, calling for more nuanced analysis on different combinations and sequences of various types of information.

^3^The upper-bound QUD inevitably includes the stronger scalar term all, as common in many QUD studies (e.g., Levinson, 2000; Politzer-Ahles and Fiorentino, 2013; Yang et al., 2018). Although a response with some is obviously underinformative with a QUD containing all, the point here is that speakers would give lower ratings in such scenario than in a lower-bound QUD.

^4^The reason why we did not include the open-ended question for every trial is that we suspect that participants’ justifications, say if a participant thinks that the L1 speaker is underinfomative due to unwillingness, would not be drastically changed in a very short amount of time. Additionally, if the same underinformative scenario and the highly identical question keep occurring in the experiment and participants are repeatedly asked to enter the same answer, we are concerned that they might be annoyed by such repetition.

^5^ www.credamo.com

^6^What remains unclear is how exactly an English mindset would influence speakers to produce underinformative utterances since participants in the current study did not elaborate on this point.

^7^However, it remains unclear whether the L1 English participants in Fairchild and Papafragou (2018) were bilingual or not, and all the participants in Fairchild et al. (2020) were monolingual speakers of English.

^8^Examples of linguistic difficulty in *ad hoc* implicature are often associated with failure to say names of objects in Chinese, e.g., "S/he does not know the word *chips* in Chinese." The accented L2 speaker was most frequently provided with such explanation (34.21%), much higher than the accent-free L2 speaker (13.51%).
