# Supplementary material for: Chinese comprehenders’ interpretation of underinformativeness in L1 and L2 accented speech narratives
Source: Front Psychol. 2023 Jan 23;14:1040162. doi: 10.3389/fpsyg.2023.1040162 (PMC9900116; doi:10.3389/fpsyg.2023.1040162)
Supplement: Supplementary file 1 [file Data_Sheet_1.PDF]

Supplementary Material

1 Supplementary Tables

Table 1. Output of the cumulative link mixed model for all the conditions in scalar implicature

Effect	Estimate	Std. Err.	*z* value	*p* value
Condition: Underinformative ‘any’ vs: Optimal	6.48	0.21	30.24	<.0001 ***
Condition: Underinformative ‘any’ vs: False	-4.20	0.30	-14.10	<.0001 ***
Condition: Underinformative ‘any’ vs: Underinformative ‘all’	-0.18	0.17	-1.05	0.29
List: A vs. B	0.71	0.33	2.11	0.03 *
Speaker: L1 vs. L2 E	0.89	0.20	4.48	<.0001 ***
Speaker: L1 vs. L2 J	1.38	0.24	5.72	<.0001 ***
Condition: Underinformative ‘any’ vs. Optimal × List: A vs. B	-0.91	0.26	-3.55	<.0001 ***
Condition: Underinformative ‘any’ vs. False × List: A vs. B	-0.73	0.42	-1.76	0.07
Condition: Underinformative ‘any’ vs. Underinformative ‘all’ × List: A vs. B	0.03	0.23	0.12	0.90
Condition: Underinformative ‘any’ vs. Optimal × Speaker: L1 vs. L2 E	-0.79	0.26	-2.99	0.002 *
Condition: Underinformative ‘any’ vs. False × Speaker: L1 vs. L2 E	-0.95	0.40	-2.35	0.019 *
Condition: Underinformative ‘any’ vs. Underinformative ‘all’ × Speaker: L1 vs. L2 E	-0.05	0.23	-0.21	0.84

Condition: Underinformative 'any' vs. Optimal ⨯ Speaker: L1 vs. L2 J	-0.88	0.27	-3.24	0.001 **
Condition: Underinformative 'any' vs. False ⨯ Speaker: L1 vs. L2 J	-0.82	0.38	-2.15	0.032 *
Condition: Underinformative 'any' vs. Underinformative 'all' ⨯ Speaker: L1 vs. L2 J	0.06	0.23	0.24	0.81
List: A vs. B ⨯ Speaker: L1 vs. L2 E	-0.14	0.28	-0.49	0.62
List: A vs. B ⨯ Speaker: L1 vs. L2 J	-0.50	0.34	-1.50	0.13
Condition: Underinformative 'any' vs. Optimal ⨯ List: A vs. B ⨯ Speaker: L1 vs. L2 E	-0.12	0.36	-0.32	0.75
Condition: Underinformative 'any' vs. False ⨯ List: A vs. B ⨯ Speaker: L1 vs. L2 E	-1.17	0.67	-1.75	0.08
Condition: Underinformative 'any' vs. Underinformative 'all' ⨯ List: A vs. B ⨯ Speaker: L1 vs. L2 E	0.17	0.32	0.52	0.60
Condition: Underinformative 'any' vs. Optimal ⨯ List: A vs. B ⨯ Speaker: L1 vs. L2 J	-0.02	0.37	-0.05	0.96
Condition: Underinformative 'any' vs. False ⨯ List: A vs. B ⨯ Speaker: L1 vs. L2 J	-0.88	0.60	-1.47	0.14
Condition: Underinformative 'any' vs. Underinformative 'all' ⨯ List: A vs. B ⨯ Speaker: L1 vs. L2 J	0.09	0.32	0.29	0.77

Note: *$p < 0.05$; **$p < 0.01$; ***$p < 0.001$.

Reference level for Condition: underinformative-"any"; Reference level for List: A; Reference level for Speaker: L1 speaker

L1 = native speaker; L2 E = accent-free L2 speaker Emma; L2 J = accented L2 speaker John

Table 2. Output of the cumulative link mixed model for all the conditions in ad hoc implicature

Effect	Estimate	Std. Err.	*z* value	*p* value
Condition: Underinformative vs: Optimal	7.20	0.28	25.79	<.0001 ***
Condition: Underinformative vs: False	-7.72	0.41	-18.94	<.0001 ***
List: A vs. B	-0.58	0.43	-1.37	0.17
Speaker: L1 vs. L2 E	0.58	0.20	2.84	0.004 **
Speaker: L1 vs. L2 J	0.91	0.25	3.61	<.0001 ***
Condition: Underinformative vs. Optimal ╳ List: A vs. B	-0.44	0.31	-1.45	0.15
Condition: Underinformative vs. False ╳ List: A vs. B	-0.60	0.60	-1.00	0.32
Condition: Underinformative vs. Optimal ╳ Speaker: L1 vs. L2 E	-0.53	0.33	-1.61	0.11
Condition: Underinformative vs. False ╳ Speaker: L1 vs. L2 E	0.04	0.49	0.08	0.94
Condition: Underinformative vs. Optimal ╳ Speaker: L1 vs. L2 J	0.10	0.38	0.27	0.79
Condition: Underinformative vs. False ╳ Speaker: L1 vs. L2 J	0.42	0.46	0.90	0.37
List: A vs. B ╳ Speaker: L1 vs. L2 E	-0.12	0.29	-0.41	0.68
List: A vs. B ╳ Speaker: L1 vs. L2 J	-0.36	0.36	-1.01	0.31
Condition: Underinformative vs. Optimal ╳ List: A vs. B ╳ Speaker: L1 vs. L2 E	0.64	0.43	1.49	0.14
Condition: Underinformative vs. False ╳ List: A	-0.31	0.79	-0.40	0.69

vs. B × Speaker: L1 vs. L2 E				
Condition: Underinformative vs. Optimal × List: A vs. B × Speaker: L1 vs. L2 J	-0.28	0.47	-0.59	0.56
Condition: Underinformative vs. False × List: A vs. B × Speaker: L1 vs. L2 J	0.84	0.72	1.16	0.24

Note: *p < 0.05; **p < 0.01; ***p < 0.001.

Reference level for Condition: underinformative; Reference level for List: A; Reference level for Speaker: L1 speaker

L1 = native speaker; L2 E = accent-free L2 speaker Emma; L2 J = accented L2 speaker John

Table 3. Output of the cumulative link mixed model for the underinformative condition in the two types of inference

Effect	Estimate	Std. Err.	*z* value	*p* value
Type: SI vs. Ad hoc	-1.76	0.14	-12.27	<.0001 ***
Speaker: L1 vs. L2 E	0.54	0.23	2.32	0.02 *
Speaker: L1 vs. L2 J	0.75	0.26	2.91	0.004 **
Type: SI vs. Ad hoc × Speaker: L1 vs. L2 E	0.49	0.21	2.32	0.02 *
Type: SI vs. Ad hoc × Speaker: L1 vs. L2 J	0.68	0.20	3.45	<.0001 ***

Note: *p < 0.05; **p < 0.01; ***p < 0.001.
Reference level for Type: scalar implicature; Reference level for Speaker: L1 speaker

SI = scalar implicature; Ad hoc = ad hoc implicature

L1 = native speaker; L2 E = accent-free L2 speaker Emma; L2 J = accented L2 speaker John

Table 4. Output of the cumulative link mixed model for the five attributes

Effect	Estimate	Std. Err.	*z* value	*p* value
Speaker: L1 vs. L2 E	1.40	0.43	3.25	0.001 **

Speaker: L1 vs. L2 J	2.15	0.45	4.73	<.0001 ***
Attribute: Honesty vs. Reliability	-0.33	0.36	-0.92	0.36
Attribute: Honesty vs. Perspective-taking	-1.29	0.37	-3.50	<.0001 ***
Attribute: Honesty vs. Becoming friends	-0.50	0.36	-1.39	0.17
Speaker: L1 vs. L2 E × Attribute: Honesty vs. Reliability	-0.30	0.49	-0.61	0.54
Speaker: L1 vs. L2 J × Attribute: Honesty vs. Reliability	-0.42	0.49	-0.86	0.39
Speaker: L1 vs. L2 E × Attribute: Honesty vs. Perspective-taking	-0.03	0.50	-0.05	0.96
Speaker: L1 vs. L2 J × Attribute: Honesty vs. Perspective-taking	0.23	0.50	0.46	0.65
Speaker: L1 vs. L2 E × Attribute: Honesty vs. Becoming friends	1.09	0.50	2.20	0.028 *
Speaker: L1 vs. L2 J × Attribute: Honesty vs. Becoming friends	0.85	0.49	1.73	0.08
Speaker: L1 vs. L2 E × Attribute: Honesty vs. Communication skills	-0.07	0.49	-0.14	0.89
Speaker: L1 vs. L2 J × Attribute: Honesty vs. Communication skills	-1.45	0.49	-2.95	0.003 **

Note: *$p < 0.05$; **$p < 0.01$; ***$p < 0.001$.

Reference level for Speaker: L1 speaker; Reference level for Attribute: Honesty

L1 = native speaker; L2 E = accent-free L2 speaker Emma; L2 J = accented L2 speaker John
